# Urinary Exosomes Identify Inflammatory Pathways in Vancomycin Associated Acute Kidney Injury

**DOI:** 10.3390/ijms22062784

**Published:** 2021-03-10

**Authors:** Linda Awdishu, Amy Le, Jordan Amato, Vidhyut Jani, Soma Bal, Robert H. Mills, Marvic Carrillo-Terrazas, David J. Gonzalez, Ashita Tolwani, Anjali Acharya, Jorge Cerda, Melanie S. Joy, Paola Nicoletti, Etienne Macedo, Sucheta Vaingankar, Ravindra Mehta, Satish P. RamachandraRao, on behalf of the Direct Investigators

**Affiliations:** 1 Skaggs School of Pharmacy and Pharmaceutical Sciences, University of California, San Diego, CA 92093, USA; a6le@ucsd.edu (A.L.); jegilmor@ucsd.edu (J.A.); vgjani@ucsd.edu (V.J.); rhmills@health.ucsd.edu (R.H.M.); mac215@health.ucsd.edu (M.C.-T.); djgonzalez@ucsd.edu (D.J.G.); 2 School of Medicine, University of California, San Diego, CA 92093, USA; sobal@ucsd.edu (S.B.); emacedo@ucsd.edu (E.M.); svaingankar@health.ucsd.edu (S.V.); rmehta@ucsd.edu (R.M.); 3 School of Medicine, University of Alabama, Birmingham, AL 35233, USA; atolwani@uab.edu; 4 Albert Einstein College of Medicine, The Bronx, NY 10461, USA; anjali2526@gmail.com; 5 Albany Medical College, Albany, NY 12208, USA; jorge.cerda82@gmail.com; 6 Division of Renal Diseases and Hypertension, Skaggs School of Pharmacy and Pharmaceutical, Aurora, CO 80045, USA; MELANIE.JOY@cuanschutz.edu; 7 School of Medicine, University of Colorado, Aurora, CO 80045, USA; 8 Mount Sinai School of Medicine, New York, NY 10029, USA; paola.nicoletti@mssm.edu; 9 Department of Cellular and Molecular Medicine, University of Michigan Medical System, Ann Arbor, MI 48109, USA; satishpr@med.umich.edu

**Keywords:** vancomycin, AKI, nephrotoxicity, exosomes, inflammation, complement, immune pathways

## Abstract

Background: Vancomycin is commonly used as a first line therapy for gram positive organisms such as methicillin resistant *Staphylococcus* *aureus*. Vancomycin-induced acute kidney injury (V-AKI) has been reported in up to 43% of patients, especially in those with higher targeted trough concentrations. The precise mechanism of injury in humans remains elusive, with recent evidence directed towards proximal tubule cell apoptosis. In this study, we investigated the protein contents of urinary exosomes in patients with V-AKI to further elucidate biomarkers of mechanisms of injury and potential responses. Methods: Urine samples from patients with V-AKI who were enrolled in the DIRECT study and matched healthy controls from the UAB-UCSD O’Brien Center Biorepository were included in the analysis. Exosomes were extracted using solvent exclusion principle and polyethylene glycol induced precipitation. Protein identity and quantification was determined by label-free liquid chromatography mass spectrometry (LC/MS). The mean peak serum creatinine was 3.7 ± 1.4 mg/dL and time to kidney injury was 4.0 ± 3.0 days. At discharge, 90% of patients demonstrated partial recovery; 33% experienced full recovery by day 28. Proteomic analyses on five V-AKI and 7 control samples revealed 2009 proteins in all samples and 251 proteins significantly associated with V-AKI (Pi-score > 1). The top discriminatory proteins were complement C3, complement C4, galectin-3-binding protein, fibrinogen, alpha-2 macroglobulin, immunoglobulin heavy constant mu and serotransferrin. Conclusion: Urinary exosomes reveal up-regulation of inflammatory proteins after nephrotoxic injury in V-AKI. Further studies are necessary in a large patient sample to confirm these findings for elucidation of pathophysiologic mechanisms and validation of potential injury biomarkers.

## 1. Introduction

Vancomycin is a glycopeptide antibiotic used for treatment of methicillin-resistant *Staphylococcus aureus* infections in critically ill patients. Nephrotoxicity is a serious adverse event impacting 10–20% of treated patients and is associated with increased hospital length of stay, 30-day hospital readmission rates and all-cause 30-day mortality [1]. Vancomycin associated acute kidney injury (V-AKI) is dose-related and with an onset of 4–17 days therapy initiation. Although V-AKI has long been recognized as a major side effect of vancomycin, the mechanisms of nephrotoxicity have remained poorly understood. The understanding of the cellular and molecular responses triggered by vancomycin is of key importance to understand mechanisms and develop strategies to minimize the risk of V-AKI and associated costs, while maximizing its efficacy in the treatment of infections. The toxic renal response appears to be caused by a direct effect of vancomycin on the epithelium of the proximal tubules [2]. To date, standard toxicogenomic approaches have been applied to study V-AKI in mouse models, but human studies are lacking [3,4,5]. Animal models demonstrate that vancomycin administration induces oxidative stress that may contribute to kidney injury [6,7]. One possible mechanism of injury is trapping of the drug in the proximal tubular cells since it is transported by the organic cation transporters (OCTs) across the basolateral membrane and into the proximal tubule cell, but no active efflux transport mechanisms have been identified [6,7,8].

Exosomes are membrane-derived microcytic vesicles that play a key role in intercellular communication and protein/nucleic acid delivery and provide the potential for discovery of novel biomarkers to aid in the clinical diagnosis of kidney injury [9]. It has been proposed that exosomes have greater specificity and sensitivity for biomarker discovery, attributed to the stability of specimens compared to transcriptomic and proteomic approaches using conventional serum and urine samples [9]. In this study we tested the hypothesis that the change in urinary exosome proteins in response to V-AKI helps elucidate mechanisms of injury and identify novel biomarkers among patients with confirmed drug-induced kidney injury. For this, we employed subjects from the Drug Induced Renal Injury Consortium (DIRECT) study, which is an international, multi-center cohort of clinically adjudicated cases of drug induced acute kidney injury and population-based controls to examine pharmacogenomics predictors using a genome wide association. Details of the DIRECT study are described in the methods section and a recent publication from our laboratory [10]. 

## 2. Results

Urine specimens from 22 subjects, 10 V-AKI cases and 12 controls, were included in the analyses. The patient demographics were similar between V-AKI cases and controls and are summarized in Table 1. As expected, the prevalence of hypertension, diabetes and heart failure was higher V-AKI cases compared to controls (Table 1).

**Figure 1:**
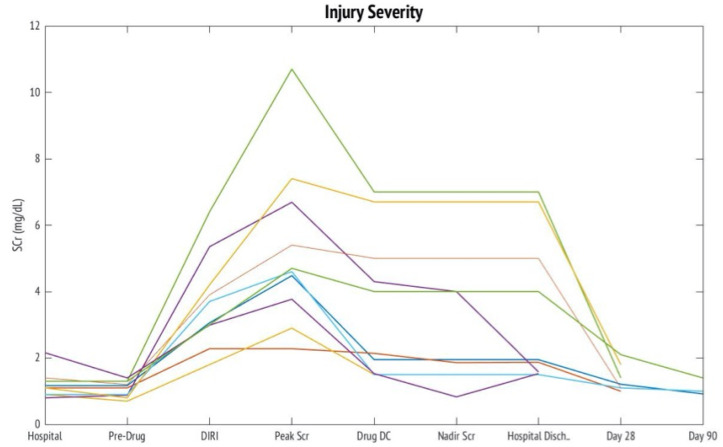
Changes in Serum Creatinine over V-AKI Course. This figure depicts the changes in serum creatinine in 10 cases over the course of vancomycin associated acute kidney injury from hospital admission through day 90 post injury. The time points correspond to Drug Induced Renal Injury Consortium (DIRECT) study time points. Abbreviations: SCr = serum creatinine, Hospital = hospital admission, Predrug = prior to drug exposure, DIRI = drug induced renal injury definition met, Drug DC = drug discontinuation or dose reduction, Nadir Scr = lowest serum after V-AKI, Hospital Disch = hospital discharge.

All V-AKI cases developed AKI stage 2 or higher as defined by the Kidney Disease Improving Global Outcomes (KDIGO) criteria (Figure 1) [11]. Onset of V-AKI occurred 4.0 ± 3.0 days after vancomycin initiation. The time course of changes in serum creatinine (Scr) following hospital admission to hospital discharge, and thereafter, are shown in Figure 1. 

**Figure 2:**
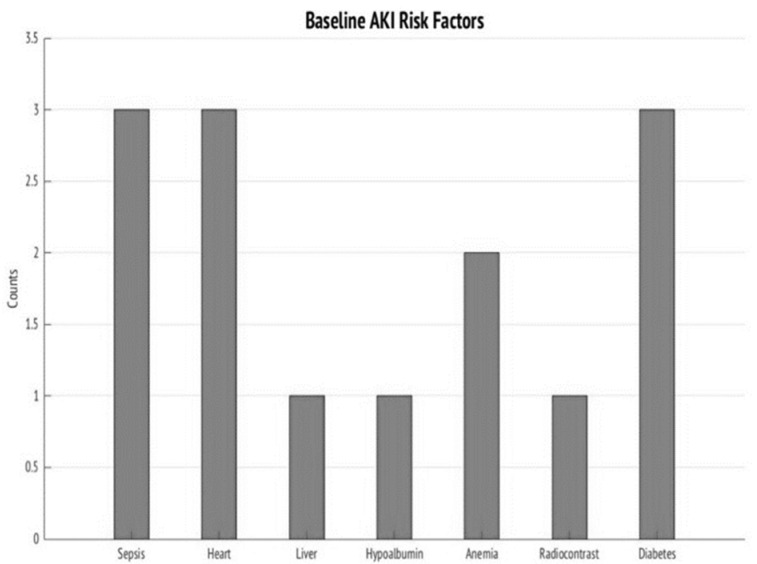
Counts of AKI Risk Factors at Baseline. This figure demonstrates the most frequent counts of AKI risk factors at baseline for V-AKI cases.

Baseline risk factors in V-AKI cases included sepsis (30%), heart disease (30%), diabetes mellitus (30%), anemia (20%), radiocontrast exposure (10%), liver disease (10%) and hypoalbuminemia (10%) (Figure 2).

**Figure 3:**
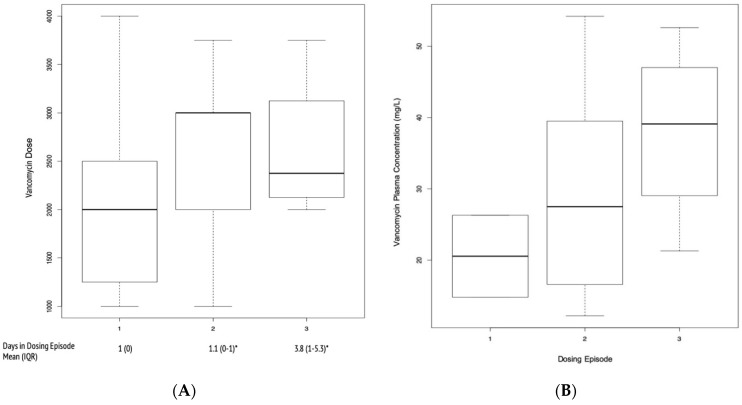
Vancomycin Daily Doses in V-AKI Cases. (**A**) A boxplot of average vancomycin daily doses (mg/day) for three dosing episodes. The mean (IQR) days spent in each dosing episode are provided. (**B**) Boxplot of vancomycin plasma concentrations (mg/L) for each dosing episode. Dark line: median, box: first and third quartiles, bars: minimum and maximum values.

At most, V-AKI cases had received three drug dosing episodes of vancomycin. Vancomycin daily doses ranged from 1000 to 4000 mg. The mean (interquartile range (IQR)) number of days spent in each dosing episode is shown in Figure 3. The mean (standard deviation (SD)) vancomycin trough plasma concentrations associated with dosing episodes 1–3 were elevated at 20.6 ± 8.1, 29.6 ± 16.3, 38.0 ± 13 mg/L, respectively. However, the difference in concentrations between each of the episodes were not statistically significant (episode 1 vs. 2 *p* = 0.5, episode 2 vs. 3 *p* = 0.41). 

Other outcomes data are summarized in Table 2. Given the severity of injury, 20% of patients required renal replacement therapy. The in-hospital mortality was 10%. Upon hospital discharge, 90% of patients did not recover from V-AKI. Six patients had Scr measured at day 28 post-injury and four at day 90. By day 28, two of six (33%) patients had AKD but none of four remaining patients had AKD by day 90. Kidney biopsies were conducted in 20% of V-AKI cases (Table 2).

### Urine Exosome Proteomic Analysis Showed Upregulation of Inflammatory Pathways

**Figure 4:**
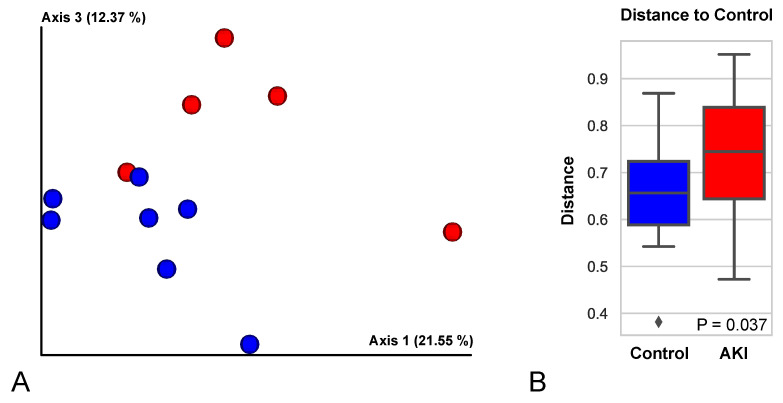
Proteome Composition Distinguishes Urinary Exosomes of V-AKI Patients and Controls. (**A**) Principal coordinates analysis of sample proteomes. The Bray-Curtis distance metric was used to calculate differences between sample proteome and a principal coordinates plot is shown. Separation along axes 1 and 3 were observed between AKI samples (red) and control samples (blue). (**B**) Boxplot summarizing Bray-Curtis distance between control and AKI samples. Statistical separation between AKI and control samples shown in (**A**) was tested using a permutational analysis of variance test (PERMANOVA). Boxplots are shown summarizing the results when comparing distances of control or AKI samples to control samples.

The 12 samples that were qualified for analyses after imposing the filter for matching the peptides with their cognate spectra, contained a total of 2009 proteins These proteins were analyzed further. First, a principal coordinates analysis (PCoA) was performed to compare the overall proteome of each sample. This analysis revealed a significant separation (PERMANOVA *p*-value = 0.037) between AKI and control samples (Figure 4), recapitulating the systemic level phenotype at the exosome protein level as well, thus increasing our confidence in these data sets. The strength of the association between each protein and V-AKI status was ranked using the pi-value metric, which combines *p*-value significance with fold-change [12]. 

**Figure 5:**
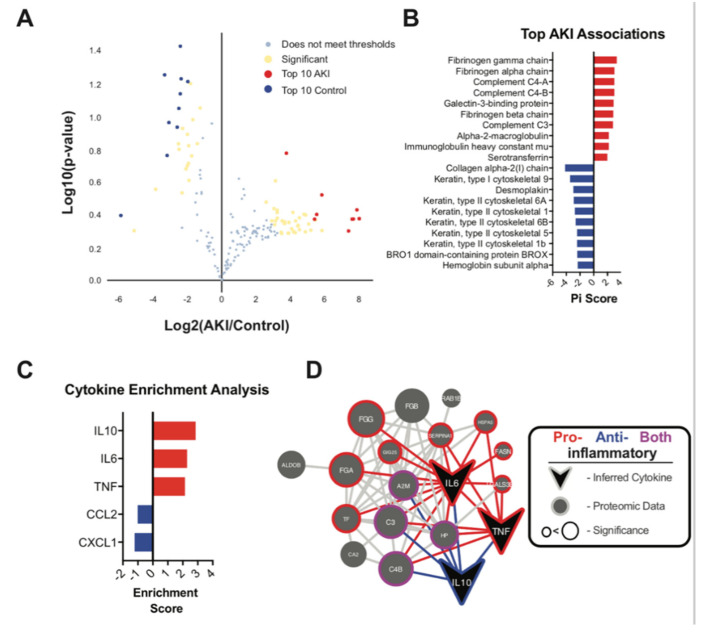
Protein-level Associations to V-AKI. (**A**) Volcano plot displaying the fold-change and *t*-test significance of each protein to V-AKI status. The top 10 proteins associated with AKI and control status are shown in blue and red respectively. Other associated proteins based on a Pi Score > 1 are shown in yellow. (**B**) A Pi score, which accounts for both fold-change and *t*-test significance was calculated for each protein and the proteins with the strongest associations are plotted. (**C**) Top cytokines associated with AKI or control proteins. A network-based cytokine inference approach was taken and the cytokines showing the strongest enrichment scores toward either AKI or control-associated proteins are shown. Red bars represent cytokines related to AKI-associated proteins, blue bars represent cytokines related to control-associated proteins. (**D**) Protein-protein interaction network of AKI-associated proteins and AKI-enriched inferred cytokines. The outer edge of protein nodes is colored dependent upon associations to pro or anti- inflammatory cytokines. The size of each node represents the strength of the statistical association of each protein to AKI status.

Next, binary comparisons between V-AKI and control samples yielded 42 proteins significantly associated with AKI phenotype and 26 proteins associated with control phenotype (Pi score > 1) (Supplemental Table S1). A volcano plot illustrating the significance and fold-change associated with each protein is shown in Figure 5A. The top discriminatory proteins for V-AKI cases were fibrinogen, complement C3, complement C4, galectin-3-binding protein, alpha-2 macroglobulin, immunoglobulin heavy constant mu and serotransferrin (Figure 5B). Gene set enrichment analysis was performed among significant proteins to identify categories of proteins altered in V-AKI patients and the results are summarized in Supplementary Table S1. Several proteins that were associated with the AKI-phenotype were shown to have functions tied to inflammation and/or mediating inflammatory phenotype. To test whether these protein changes might be related to changes in underlying cytokine signaling, a cytokine inference was performed, which indicated a strong relationship between AKI-associated proteins and cytokines IL10, IL6 and TNF. These cytokines contained 2.8, 2.3 and 2.1-fold more connections to AKI-associated proteins than control-associated proteins, respectively, as summarized in Figure 5C. Protein-protein interaction networks of AKI-associated proteins and inferred cytokines showed predominately proinflammatory connections among AKI-associated proteins as summarized in Figure 5D.

## 3. Discussion

We report a novel approach to utilize urinary exosomes to inform pathophysiological mechanisms involved in drug-induced kidney injury using vancomycin as the prototypical renal toxicant. One of the strengths of this study is the incorporation of well-characterized patient-level data from the DIRECT study [10] developed to elucidate mechanisms including genomics of drug-induced kidney injury. Subjects with stage 2 or higher AKI were exposed to conventional risk factors at baseline. All study subjects had significantly elevated vancomycin plasma trough concentrations despite receiving daily doses less than the generally accepted nephrotoxicity threshold of 4 g per day [13]. Many subjects did not completely recover from V-AKI at hospital discharge and had acute kidney disease for the month following injury. As expected, few patients received a kidney biopsy. 

Urinary exosome/extracellular vesicle (EV) proteomics demonstrated that the targeted discriminatory proteins for V-AKI cases included C3 complement, C4 complement, galectin-3-binding protein, fibrinogen, alpha-2 macroglobulin, immunoglobulin heavy constant mu and serotransferrin. These exosome findings inform mechanisms of injury at sub-cellular/supra-molecular level for V-AKI and potentially serve as biomarkers along the continuum of the drug induced disease process.

Exosomes, present in blood and urine, have important roles in intercellular communication, coagulation and waste management [14]. After toxin-related acute kidney injury, exosomes released from tubular epithelial cells may communicate injury signals to recruit macrophage infiltration and promote tubulointerstitial inflammation [15]. We reasoned that the protein contents of exosomes would differ between V-AKI cases and healthy controls. Two hundred and fifty-one proteins were dysregulated in V-AKI, and these appeared to be predominantly involved in the inflammatory and coagulation pathways. Among these 251 proteins, our analysis demonstrated that C3 complement, C4 complement, galectin-3-binding protein, fibrinogen, alpha-2 macroglobulin, immunoglobulin heavy constant mu and serotransferrin were significantly associated with V-AKI cases. 

Based on our results that C3 and C4 were among the top discriminating V-AKI exosome proteins, we propose that activation of a complement system is involved in vancomycin-induced renal tubular injury. This is also in good agreement with published literature that the complement system activation plays a role in immune complex glomerulonephritis and a diverse array of kidney diseases [16]. Complement activation-mediated kidney damage in AKI due to ischemia or nephrotoxin exposure has been linked to systemic inflammatory events that trigger and contribute to remote organ injury and patient mortality [17]. In models of ischemia-reperfusion (IR) injury, hypoxia, ATP depletion and mitochondrial damage result in the generation of free oxygen radicals upon reperfusion [18]. Cytokines, chemokines and complement activation amplify the inflammation, leading to acute tubular injury. Additionally, C3a and C5a receptor deficient mice are protected from IR injury [19,20]. IR injury has been shown to upregulate local production of complement components by kidney endothelial and tubular cells [16,21]. Several studies in animal models and human subjects have identified increased deposition of complement protein products such as C3 and C4 along the tubular basement membrane after trauma or injury in the kidney. These increased levels of depositions have not been found to be present significantly in the peritubular or glomeruli region, but are concentrated heavily in the tubular membrane, as seen in biopsies of human and animal model kidneys [17]. Exosomes and EVs can transport circulatory complement components when complement activation is dysregulated [22,23]. Several cellular experiments and clinical observations have confirmed that released EVs carry and modulate the complement proteins and, simultaneously, their production under an inflammatory condition is also able to influence the number of circulatory vesicles [23]. This interdependence of the two processes leads to the conclusion that EVs exhibit the potential of being biomarker sources, targets and therapeutic delivery agents toward complement and immunomodulatory compounds that are highly relevant in the scope of inflammatory mechanisms of AKI. Our finding that the increased concentrations and presence of complement proteins C3 and C4 in the EVs of V-AKI samples compared to healthy controls, demonstrates a role of immune responses to direct toxicity of vancomycin in kidney tubular cells. In addition to all the above protein content, exosomes/EVs derived from urine also represent potential noninvasive biomarkers as alternatives to kidney biopsy to detect and inform about the role of the complement system in drug-induced kidney injury.

Although many previous studies (see next few sentences for specific references) have focused on the role of galectin-3 (Gal-3) in kidney injury, there is little data on the galectin-3 binding protein (Gal-3-bp). Gal-3 is expressed in the collecting tubules of the kidney and functions to promote nephrogenesis, modulate cell-cell and cell-matrix interactions, and regulate inflammation [24,25,26]. Henderson et al. reviewed the role of Gal-3 in inflammation and found that Gal-3 is a key component in chronic inflammation and if tissue injury becomes recurrent. It facilitates the “walling off” of tissue injury, delaying the spread of the injury [27]. Additionally, Nishiyama et al. demonstrated in rats with ischemic AKI that Gal-3 mRNA was upregulated and following reperfusion, immunohistochemistry revealed increased Gal-3 across nephron segments [25]. Tsuchiyama et al. found that administering Gal-3 in rats with nephrotoxic serum nephritis led to a reduction in urinary protein excretion, crescent formation and decreased infiltration of macrophages into glomeruli [28]. This suggests that after ischemic or immune-mediated injury, Gal-3 plays a role in regeneration. As the cognate partner to Gal-3, the fundamental role of Gal-3-bp, and the reason for its upregulation in nephrotoxic injury due to vancomycin and other agents, would be an imperative focus in future investigations. 

Our study also found that all three subunits of fibrinogen to be dysregulated, in good agreement with the previously published data. Fibrinogen is a soluble dimeric plasma molecule and each dimer is composed of three polypeptides: alpha, beta, and gamma [29]. It plays a key role in hemostasis and coagulation but has also been shown to be important in regulation of inflammatory-mediated conditions [30,31,32]. Ajay et al. showed that fibrinogen availability is important for kidney function, inflammation, and survival [33]. Further, fibrinogen might have an antiadhesive effect on kidney epithelial cells that may be beneficial by helping to prevent tubular obstruction [34]. Fibrinogen is significantly increased and upregulated in AKI and has been previously evaluated as a potential biomarker for early AKI detection [29]. Hoffman et al., studied the change in fibrinogen after ischemia/reperfusion and cisplatin administration in rats and mRNA was upregulated 30 min after bilateral IR injury and reached a peak at 48–72 h, whereas in cisplatin administration, mRNA expression of fibrinogen was detected at 24 h [29]. Urinary fibrinogen was detected as early as 3 h post ischemic-reperfusion injury and 24 h post cisplatin administration corresponding with large increases in kidney injury molecule 1 (KIM-1) [29]. 

Historically, two different pathophysiological mechanisms for V-AKI have been generally accepted, acute tubular necrosis from direct toxic effects and acute interstitial nephritis. The renal proximal tubular epithelium undergoes loss of cytoskeletal integrity, necrosis and apoptosis in acute tubular necrosis [35]. Necrotic cells release molecules which upregulate the innate immune system inducing inflammation and accelerating tubular injury [35]. In the current study, we demonstrated inflammatory markers in the urinary EVs of patients with V-AKI. Additionally, we found a proinflammatory relationship between cytokines IL10, IL6 and TNF and our top AKI discriminating proteins. Taken together, these data suggest that the pathophysiology of V-AKI is one of tubular toxicity with upregulation of inflammation during the period of 24–72 h post injury. 

There are several limitations of our study. The small sample size of 22 patients who were male and predominantly Caucasian limits the generalizability of our findings to other genders, races and ethnicities. A second limitation to our study was that each patient’s urinary exosome sample had varying amounts of total protein, thus making measurement of specific proteins difficult, especially for those of lower absolute quantities. While this is not necessarily the limitation of this study per se, as every individual’s ability to pack proteins on to their urine exosomes is naturally variable, the lower quantities of proteins in some individuals limit our ability for further analyses and drawing meaningful conclusions that are generalizable to larger populations. A third limitation is the single time point sample which limits our understanding of cellular changes occurring at different phases of the AKI continuum, e.g., immediate injury phase, repair phase, etc. Sequential sampling will further our understanding of the time-course of exosome content changes, oxidative stress induced by drug accumulation, and upregulation of the immune pathways in repair. The current study included patients as defined by Scr thresholds. Serum creatinine is an imperfect kidney functional biomarker since elevations in Scr often lag behind the injury [36]. This limits the sensitivity and specificity of Scr to detect early kidney injury [36], and studies have emerged identifying kidney injury biomarkers such as kidney injury molecule 1 (KIM1) and neutrophil gelatinase associated lipocalin (NGAL) for early detection of nephrotoxicity [37,38]. As previously reported, biomarkers have the potential for detecting both overt and sub-clinical levels of drug-induced kidney injury [39,40].

## 4. Materials and Methods

### 4.1. Patient Selection

Urine samples from 10 patients with V-AKI, who were enrolled in the DIRECT study [10] and 12 healthy control urine samples from the UAB-UCSD O’Brien Center Biorepository were included in the analysis. Control subjects were matched by gender, race, and decade of age to the study subjects and had no exposures to vancomycin or known kidney injury. The DIRECT study is an international study examining the pharmacogenetic predictors of drug induced kidney injury using a genome wide association study of cases and population-based controls [10]. The DIRECT study was approved by the institutional research board (IRB #121651) and written informed consent was obtained from all participants prior to enrollment which included the provision of utilizing stored specimens for future analyses. AKI was defined in DIRECT as an abrupt reduction in kidney function demonstrated by any of the following criteria: (1) absolute increase in serum creatinine (Scr) (≥0.3 mg/dL or ≥26.4 µmol/L) (within 48-h time window) from the reference Scr value, (2) percentage increase in Scr of ≥50% (1.5 fold from reference) within seven days, (3) reduction in urine output (documented oliguria of <0.5 mL/kg/h for >6 h) despite adequate fluid resuscitation when applicable, (4) absolute decrease in Scr of ≥0.3 mg/dL or ≥26.4 µmol/L (within 48-h time window) from the reference Scr, and (5) relative decrease in Scr of ≥50% (1.5 fold from reference) within seven days. Patients with stage 2 AKI or higher following vancomycin exposure were enrolled in the study. Clinical data including demographics, medical history, physical exam findings, vital signs, blood and urine chemistries, biopsy results and drug dosing data, were collected at the following time points: (1) hospital admission, (2) initiation of vancomycin, (3) peak of injury, (4) discontinuation of the drug or dose reduction, (5) injury resolution, (6) hospital discharge, (7) 28 days- and (8) 90 days-post injury. A vancomycin dosing episode was defined as a change in the dose or frequency. Acute kidney disease (AKD) is defined as persistence of stage 1 AKI or greater ≥7 days after exposure [41]. Patients were categorized with AKD at hospital discharge if AKI stage 1 or greater persisted and the time from exposure to hospital discharge was greater than seven days. Urine and whole blood samples were obtained from all participants. All cases of V-AKI were adjudicated by two independent nephrologists (EM, JC, RL) to determine causality. A full description of the methods has been previously published [10]. 

### 4.2. Exosome Preparation and Proteomic Data Collection

Urine and blood samples were collected from DIRECT study participants and samples were frozen at −80 °C prior to exosome preparation. Urine samples used in this analysis were selected from the time period closest to kidney insult ranging from 24 to 72 h post kidney injury. Exosomes were extracted from frozen AKI urine samples using an in-house protocol developed based on the solvent exclusion principle using polyethylene glycol (PEG)-induced precipitation, as described in the Rao et al. recent publication [42]. SDS-page of the exosomal proteins was done prior to gel trypsinization using NuPAGE bisTris 10% acrylamide 12 well gels to resolve 40 μL of protein from exosomes of the control and V-AKI urine samples. 

Gel extracts were combined with a buffer to prevent proteolysis which contained 75 mM NaCl (Sigma, St. Louis, MO, USA), 3% sodium dodecyl sulfate (SDS, Fischer, Arnold AFB, Tullahoma, TN, USA), 1 mM NaF (Sigma, St. Louis, MO, USA), 1 mM beta-glycerophosphate (Sigma, St. Louis, MO, USA), 1 mM beta-glycerophosphate (Sigma, Sigma, St. Louis, MO, USA), 1 mM sodium orthovanadate (Sigma, St. Louis, MO, USA), 10 mM sodium pyrophosphate (Sigma, St. Louis, MO, USA), 1 mM phenylmethylsulfonyl fluoride (PMSF, Sigma), and 1× Complete Mini EDTA free protease inhibitor tablet all in 50 mM HEPES (Sigma, St. Louis, MO, USA), pH 8.5. To denature proteins, an equal volume 8 M Urea in 50 mM HEPES, pH 8.5 was added and probe sonication was performed using two 10-s intervals at 25% amplitude. Proteins were reduced, alkylated and quenched using dithiothreitol and iodoacetamide, as previously described [43]. Proteins were precipitated by adding trichloroacetic acid (Sigma, St. Louis, MO, USA) to samples on ice, and pelleting proteins through centrifugation at 4 °C. Protein pellets were washed twice with ice-cold acetone. Protein pellets were dried at 56 °C for 30 min, then resuspended in 1 M urea in 50 mM HEPES then digested overnight at room temperature with 6 μg of LysC (Wako, Richmond, Commonwealth of Virginia, USA) followed by a 6-h digestion with sequencing-grade trypsin (Promega, Fitchburg, WI, USA) at 37 °C. Samples were desalted through C18 Sep-Paks and proteins were quantified [44]. A total of 1.0 ug of protein per sample was analyzed by liquid-chromatography tandem mass spectrometry (LC-MS [2]) using an 85 min liquid chromatograph gradient on an Easy-nLC 1000 (Thermo Fischer Scientific, Waltham, MA, USA) connected in line to a Orbitrap Fusion mass spectrometer (Thermo Fischer Scientific). 

Chromatography was performed on a home-pulled and packed 30 cm column was triple-packed with 0.5 cm, 0.5 cm and 30 cm of 5 μm C4, 3 μm C18, and 1.8 μm C18, respectively, and heated to 60 °C. Peptides were first loaded at 500 bar which was followed by a chromatography gradient ranging from 6 to 25% acetonitrile over 70 min followed by a 5-min gradient to 100% acetonitrile, which was held for 10 min. Electrospray ionization was performed by applying 2000 V through a stainless-steel T-junction connecting the analytical column and Easy-nLC system. Each sample was followed by two washes starting with a gradient from 3 to 100% acetonitrile over 15 min with an additional 10 min at 100% acetonitrile. A mass to charge (*m/z*) range of 375–1500 was scanned for peptides with charge states between 2–6. Centroided data was used for quantitation of peaks. Acquisition was run in a data-dependent positive ion mode. Raw spectra was searched in Proteome Discoverer Version 2.1 against the uniprot (www.uniprot.org (accessed on 5 November 2017)) reference database for *Homo sapiens* using the Sequest algorithm [18] alongside a reverse database approach used to control peptide and false discovery rates (FDR) to 1% [17]. In silico trypsin digest was specified in the search parameters alongside a minimum peptide length of six amino acids. Search settings also included dynamic modification for oxidation of methionines and a static modification of carbamidomethylation of cysteines. Precursor mass tolerance was set to 50 ppm and fragment mass tolerance of 0.6 Da.

In addition to spectral matches and proteins being restricted to <1% FDR, peptide matches were further screened to only include peptide spectral matches (PSMs) identified with high confidence. The summed abundance of the area under the curve of MS1 peaks associated with PSMs were used to represent protein abundances. To help account for run-to-run variation in LC-MS2 experiments, the raw protein abundances were next normalized by a two-step process. In the first step, protein abundances were divided by the average abundance of a given protein throughout the experiment divided by the median of the protein averages observed throughout the experiment. In the second step, the adjusted protein abundances were divided by the median protein abundances observed within their respective samples divided by the median protein abundance observed in the entire experiment. Records of the normalization and analysis of proteomics data are available in a jupyter notebook form at github.com (https://github.com/rhmills/VAKI_Exosome_Proteomics (accessed on 3 March 2021)). 

### 4.3. Analysis of Proteomics Data

Records of the proteomics data analysis were uploaded in a jupyter notebook format to a github repository (https://github.com/rhmills/VAKI_Exosome_Proteomics (accessed on 3 March 2021)). Principle coordinates analysis (PCoA) was performed using Qiime2 (version 2019-7) [45]. The qiime diversity core-metrics command was used to calculate between sample distances using the Bray-Curtis metric and visualization using a PCoA. Statistical analysis of the distances between control and V-AKI samples was performed using a pair-wise PERMANOVA test through the command “qiime diversity beta-group-significance”. A boxplot visualization of these results was created through the seaborn package (https://seaborn.pydata.org/# (accessed on 3 March 2021)). Binary comparisons of V-AKI cases and control subjects were performed using the scipy package (www.scipy.org (accessed on 3 March 2021)) using an independent *t*-test with unequal variance. The strength of the association between each protein and V-AKI status was ranked using the pi-value metric which combines *p*-value significance with fold-change [12]. As previously described [46] top ranked, significant associations were defined as having a pi-value > |1|. Each protein association to V-AKI status was plotted in a volcano plot to further highlight the location of the top-10 ranked proteins. Gene set enrichment analysis was performed using the DAVID server [47], comparing significant proteins (pi-value > |1|) to a background of all proteins identified in the dataset. Clusters of related functional enrichments are reported in Supplementary Table S1. 

To test for a relationship between V-AKI associated proteins and inflammatory cytokines, a recently developed network-based cytokine inference approach was utilized [48]. Briefly, a list of cytokines (TGFb, TNF, IFN, IL1-40, CXCL1-16, CCL1-27) was submitted alongside significantly altered proteins (pi-value > |1|) to the STRING-db tool [49]. Connections between proteins were determined using all interaction sources and a minimum interaction score >0.4. Cytokines were first filtered to have at least five connections to significantly altered proteins. Then, the proportion of connections between each cytokine and V-AKI-associated or control-associated proteins was compared with the total number of connections between V-AKI or control-associated proteins. To account for differences in the number of connections each cytokine has to other proteins, this value was next compared to the number of connections each cytokine had to all significantly altered proteins. The enrichment scores for selected cytokines were plotted for their associations with either V-AKI- or control-associated proteins. Finally, a refined search of V-AKI-associated proteins and the cytokines with enriched connections to V-AKI-associated proteins were visualized for their interactions using cytoscape version 3.5.1 [50]. Protein-protein interaction networks were decorated by sizing each V-AKI associated protein according to their pi-score association to V-AKI status, and colored by having an inferred connection to the enriched cytokines with pro or anti- inflammatory effects. Raw proteomic data files are publicly accessible at https://massive.ucsd.edu under study ID MSV000086053 (accessed on 3 March 2021).

## 5. Conclusions

We sought to better understand the mechanism of injury for V-AKI from clinically adjudicated cases and, interestingly, we found complement, galectin-3 binding protein and fibrinogen were significantly associated with V-AKI. Results of previous studies and ours suggests a role of the complement system and inflammatory pathways in V-AKI. While larger studies are needed to validate the molecular processes in the damage induced by vancomycin and the ensuing repair pathways, the results indicate that urinary exosomes may contribute important information on pathophysiologic mechanisms and may serve as biomarkers for drug-induced kidney injury.

## Figures and Tables

**Table 1:** Demographics of Vancomycin-induced Acute Kidney Injury (V-AKI) Cases and Controls.

Variable	V-AKI Cases *N* = 10	Controls *N* = 12	*p*-Value
Age (years), mean ± SD	40.8 ± 18.3	32.7 ± 14.0	0.25
Male gender, N (%)	10 (100)	12 (100)	1
Race/ethnicity, N (%) Caucasian African American Asian Hispanic American Indian or Alaska Native	6 (60) 2 (20) 1 (10) 1 (10) 0 (0)	2 (17) 2 (17) 3 (25) 0 (0) 5 (42)	0.04 0.03 0.37 0.27 0.02
Body mass index, mean ± SD	26.6 ± 7.0	25.5 ± 4.7	0.67
Comorbidities, N (%) Hypertension Diabetes mellitus Chronic obstructive pulmonary disease Coronary artery disease Heart Failure Liver disease Kidney disease Malignancy	3 (30) 3 (30) 1 (10) 2 (20) 3 (30) 1 (10) 2 (20) 1 (10)	0 (0) 0 (0) 0 (0) 0 (0) 0 (0) 0 (0) 0 (0) 0 (0)	0.05 0.05 0.27 0.11 0.05 0.27 0.11 0.27
Systolic Blood Pressure (mm Hg), mean ± SD	121.1 ± 26.9	137.1 ± 10.8	0.07
Diastolic blood pressure (mm Hg), mean ± SD	78.5 ± 15.1	76 ± 11.1	0.66

Abbreviations: Scr—serum creatinine; mm Hg—millimeters of mercury; NR—not reported.

**Table 2:** Kidney Outcomes after V-AKI. * Scr was measured in six6 patients at 28 days post injury and four patients at three months post injury. Abbreviations: AIN—allergic interstitial nephritis; AKD—acute kidney disease; ATN—acute tubular necrosis; GN—glomerulonephritis; RRT—renal replacement therapy.

Outcome	V-AKI Cases *N* = 10
Mortality, N(%)	1 (10)
Need for RRT, N(%)	2 (20)
AKI at Hospital Discharge	9 (90)
AKD at Day 28 *	2 (33)
AKD at Day 90 *	0 (0)
Kidney Biopsy, N(%)	2 (20)
AIN	1 (10)
ATN	2 (20)
GN	1 (10)

## Data Availability

Raw proteomic data files are publicly accessible at https://massive.ucsd.edu (accessed on 1 March 2021) under study ID MSV000086053.

## Supplementary Materials

The following are available online at https://www.mdpi.com/1422-0067/22/6/2784/s1.
